# Perceptions of non-alcoholic fatty liver disease – an Asian community-based study

**DOI:** 10.1093/gastro/gov047

**Published:** 2015-10-12

**Authors:** George B.B. Goh, Clarence Kwan, Sze Ying Lim, Nanda KK Venkatanarasimha, Rafidah Abu-Bakar, Thinesh L Krishnamoorthy, Hang Hock Shim, Kiang Hiong Tay, Wan Cheng Chow

**Affiliations:** ^1^Department of Gastroenterology & Hepatology, Singapore General Hospital, Singapore,; ^2^Duke-NUS Graduate Medical School, Singapore and; ^3^Department of Diagnostic Radiology, Singapore General Hospital, Singapore

**Keywords:** non-alcoholic fatty liver disease, epidemiology, perceptions, Singapore

## Abstract

**Background and Aims:** Non-alcoholic fatty liver disease (NAFLD) is the most common chronic liver disease and is closely related to metabolic syndrome and its risk factors. Worldwide, epidemiological studies have reported NAFLD prevalence rates of 5% to 30% depending on geographical variations. While epidemiological data suggest a progressively increasing prevalence of metabolic risk factors in Singapore, there are limited data about NAFLD *per se* in the community. We aim to explore the prevalence and perceptions of NAFLD in Singapore.

**Methods:** Attendees at a gastroenterology public forum were enrolled in a cross-sectional observational study evaluating demographic, anthropometric and clinical information. The diagnosis of NAFLD was based on sonographic criteria. Metabolic syndrome was defined according to International Diabetes Federation guidelines. Perceptions of NAFLD were explored using a self-administered survey questionnaire.

**Results:** A total of 227 subjects were recruited, with NAFLD being diagnosed in 40% of the cohort. Relative to those without NAFLD, subjects with NAFLD had higher male preponderance, older age, higher body mass index, waist circumference and more metabolic syndrome (all *P* < 0.05). Although 71.2% subjects had heard about NAFLD before, only 25.4% of them felt that they were at risk of NAFLD. Comparable responses were observed in subjects with no metabolic risk factors relative to subjects with one or more metabolic risk factors (*P* > 0.05). Of note, 75.6% of subjects with one or more metabolic risk factors did not think that they were at risk of NAFLD.

**Conclusion:** Our study suggests a significant local prevalence of NAFLD in the community including non-obese individuals. Considering the tendency to underestimate risk of NAFLD, enhanced public education about NAFLD is warranted to improve understanding.

## Introduction

Non-alcoholic fatty liver disease (NAFLD) is a chronic liver disease characterised by fatty infiltration in the liver which can manifest in a spectrum of diseases ranging from benign steatosis to non-alcoholic steatohepatitis (NASH) to advanced fibrosis, cirrhosis and hepatocellular carcinoma [1,2]. Because NAFLD is closely associated with metabolic syndrome and obesity, it is not unexpected that its prevalence is increasing in a manner parallel with the burgeoning obesity epidemic [3,4]. NAFLD is recognised as one of the major causes of chronic liver disease worldwide, with disease prevalence reported between 10% and 30% of the general population [5]. Of more concern, NALFD is predicted to be the leading cause of end-stage liver disease requiring liver transplantation by the year 2020 [6]. This takes on additional urgency in Asia where the most rapid increase in diabetes mellitus is being experienced (diabetes mellitus being a key component of metabolic syndrome and NAFLD) [7]. Furthermore, compared with their Western counterparts, Asian patients experience correspondingly higher rates of metabolic syndrome complications at similar levels of obesity [8,9]. There remain limited epidemiological data on NAFLD in Singapore. Based on increasing prevalence of obesity (10.8%), diabetes (11.3%), hypertension (23.5%) and dyslipidaemia (17.4%) as reported by the National Health Survey in 2010, the prevalence of NAFLD in Singapore would intuitively also be expected to be significant [10]. This would have wide-ranging clinical implications not only in clinical management but also from a public health perspective. We sought to conduct a cross-sectional study to ascertain the scale and extent of NAFLD in the Singapore general population. In addition, we explored the awareness and perceptions of NAFLD in the community.

## Methods

### Study design and population

This was an observational cross-sectional study conducted at a one-day public forum on digestive disease presently annually in Singapore by the National Foundation for Digestive Disease (NFDD). Subjects aged 21 years and older were recruited from this public forum (held on 4 November 2012) with the theme of lifestyle and digestive disease. Subjects with known chronic liver disease such as viral hepatitis B and C, alcohol-related liver disease and NAFLD were excluded from the study. Approval for the study was obtained from the Singhealth institutional review board in accordance with the ethical guidelines of the Declaration of Helsinki.

### Data collection

All consecutive subjects who agreed to participate in this study were interviewed to obtain information on demographics, medical history, job activity and exercise habits. Age, sex, ethnicity and anthropometric measurements including height, weight, body mass index (BMI) and waist circumference were recorded. Measurement of waist circumference was taken at a level midway between the lowest rib and the iliac crest, in accordance with the World Health Organisation recommendation [11]. Similarly, metabolic syndrome risk factors such as diabetes mellitus, hypertension and dyslipidaemia were explored from the medical history. Central obesity was defined based on waist circumference more than 90 cm and 80 cm in males and females, respectively. Diagnosis of metabolic syndrome was determined using the International Diabetes Federation criteria [12]. Ultrasonography was performed by a trained ultrasonographist using Philips CX50 and IU22 ultrasound machines. The diagnosis of NAFLD was ascertained by the ultrasonographist, based on standard diagnostic criteria of ‘bright’ liver echoes in the hepatic parenchyma as compared with renal parenchyma and blurring or narrowing of hepatic vessels [13]. A brief survey questionnaire was developed to assess awareness and attitudes towards NAFLD and was distributed among study subjects for self-administration.

### Statistical analysis

Descriptive statistics were computed for all variables with continuous variables expressed as means and standard deviations, while categorical variables were expressed as frequencies and percentages. Baseline characteristics stratified by presence of NAFLD were explored using the Student *t* test and Pearson’s chi-square test for continuous and categorical variables, respectively. SPSS version 21 (Chicago, Illinois, USA) statistical software package was used to conduct the statistical analysis. All *P* values quoted were two-sided with *P* < 0.05 considered statistically significant.

## Results

### Demographics and characteristics

A total of 227 subjects were recruited for this study of which 188 subjects had complete demographic and clinical information. Forty percent of the cohort had ultrasound evidence of NALFD. Table 1 illustrates the demographic, anthropometric and clinical information of the cohort, both overall and stratified according to the presence of NAFLD. The study cohort was predominantly of Chinese ethnicity, middle age (mean age of 54 years) and non-obese (mean BMI 22.7 kg/m^2^), with variable extents of metabolic syndrome risk factors. Compared with subjects without NAFLD, subjects with NAFLD had a higher male distribution, were older and had higher BMI, waist circumference and recorded blood pressure. Ethnicity, alcohol consumption and job activity were comparable between the two groups. In the context of metabolic syndrome risk factors, a higher proportion of NAFLD subjects had diabetes mellitus, central obesity and metabolic syndrome relative to the subjects without NAFLD. However, rates of hypertension and dyslipidaemia were comparable between the two groups.

**Table 1.** Demographic and clinical characteristics

Variable	Overall cohort (N = 188)	NAFLD (N = 76)	No NAFLD (N = 112)	*P* values
Male sex	79 (42.0)	41 (53.9)	38 (33.9)	0.007
Ethnicity				0.362
• Chinese	180 (95.7)	74 (97.4)	106 (94.6)	
• Indian	5 (2.7)	2 (2.6)	3 (2.6)	
• Others	3 (1.6)	0	3 (2.6)	
Age, years	54.0 ± 14.6	57.4 ± 11.6	51.9 ± 1 5.9	0.012
Body mass index, kg/m^2^	22.7 ± 3.4	24.4 ± 3.6	21.6 ± 2.6	<0.001
Waist circumference, cm				
• Male	86.3 ± 9.8	89.0 ± 9.3	83.6 ± 9.5	0.012
• Female	79.0 ± 9.6	82.2 ± 8.2	77.5 ± 9.9	0.016
Systolic blood pressure, mmHg	136 ± 22	143 ± 22	132 ± 21	<0.001
Diastolic blood pressure, mmHg	77 ± 11	81 ± 11	75 ± 11	<0.001
Alcohol consumption				0.822
• None	128 (68.1)	55 (72.4)	73 (65.2)	
• Infrequent special occasions	43 (22.9)	15 (19.7)	28 (25.0)	
• Less than once a week	8 (4.3)	3 (3.9)	5 (4.5)	
• 2–3 times per week	5 (2.7)	1 (1.3)	4 (3.6)	
• Daily	4 (2.1)	2 (2.6)	2 (1.8)	
Job activity				0.267
• Sedentary	67 (36.2)	32 (43.2)	35 (31.5)	
• Non-sedentary	48 (25.9)	17 (23.0)	31 (27.9)	
• Retiree	70 (37.8)	25 (33.8)	45 (40.5)	
Diabetes mellitus	13 (6.9)	9 (11.8)	4 (3.4)	0.027
Dyslipidaemia	66 (34.7)	30 (39.5)	36 (31.6)	0.263
Hypertension	46 (24.5)	24 (31.6)	22 (19.6)	0.059
Central obesity	77 (41.0)	42 (55.3)	35 (31.3)	0.001
Metabolic syndrome	32 (17.0)	19 (25.0)	13 (11.6)	0.014

Data expressed as mean ± standard deviation or number (%). *P* values are derived using Pearson’s chi-square tests and Student *t* tests for categorical and continuous variables, respectively. Missing data were excluded from analysis. NAFLD: non-alcoholic fatty liver disease

**Table 2.** Aspects of attitude

Questions		Overall cohort	No metabolic risk factor	≥1 metabolic risk factor	*P* value	Metabolic syndrome	No metabolic syndrome	*P* value
Have you ever heard of NAFLD before today?	Yes	126 (71.2)	38 (63.3)	58 (70.7)	0.13	22 (91.7)	74 (62.7)	0.02
	No/DK	51 (28.8)	22 (36.7)	24 (29.3)		2 (8.3)	44 (37.3)	
Is NAFLD common in Singapore?	Yes	71 (40.1)	21 (35.0)	31 (37.8)	0.71	10 (41.7)	42 (35.6)	0.66
	No/DK	106 (59.9)	39 (65.0)	51 (62.2)		14 (58.3)	76 (64.4)	
Do you think NAFLD can be a dangerous condition?	Yes	119 (68.8)	38 (65.5)	55 (68.8)	0.72	17 (70.8)	76 (66.7)	0.69
	No/DK	54 (31.2)	20 (34.5)	25 (31.2)		7 (29.2)	38 (33.3)	
Do you think you are at risk of NAFLD?	Yes	45 (25.4)	10 (16.7)	20 (24.4)	0.30	9 (37.5)	21 (17.8)	0.03
	No/DK	132 (74.6)	50 (83.3)	62 (75.6)		15 (62.5)	97 (82.2)	
Would you undergo medical screening for NAFLD?	Yes	129 (73.7)	37 (61.7)	64 (79.0)	0.04	19 (82.6)	82 (69.5)	0.31
	No/DK	46 (26.3)	23 (38.3)	17 (21.0)		4 (17.4)	36 (30.5)	

Data expressed as number (%). *P* values are derived using Pearson’s chi-square tests for categorical. Missing data were excluded from analysis. NAFLD: non-alcoholic fatty liver disease;.

### Attitudes

One hundred and twenty-six (71.2%) subjects had heard about NAFLD before, with 40.1% of them believing that NAFLD was common in Singapore. However, only 25.4% of subjects felt that they were at risk of NAFLD. The majority of subjects (68.8%) felt that NAFLD can be a dangerous condition, and 73.7% of the cohort indicated they would undergo medical screening for the disease. Attitudes were subsequently explored according to presence of metabolic risk factors and established metabolic syndrome. In general, comparable responses were observed in subjects with no metabolic risk factors relative to subjects with one or more metabolic risk factors. Of note, 75.6% of subjects with one or more metabolic risk factors did not think they were at risk of NAFLD. However, when metabolic syndrome (central obesity plus two other metabolic risk factors) was considered, significantly more subjects with metabolic syndrome had heard about NAFLD before (91.7% *vs* 62.7%, *P* = 0.02) and felt that they were at risk of NAFLD (37.5% *vs* 17.8%, *P* = 0.03).

## Discussion

Our present community-based study explored the basic epidemiology and attitudes towards NAFLD in a developed Asian country. At 40% of the study cohort, our study suggests a significant frequency of NAFLD in the community, which is similar to other reported population-based Western and Eastern studies. Utilizing ultrasound or magnetic resonance spectroscopy (MRS) modalities, population-based studies from the USA have observed prevalence rates between 19.5% and 46%, while reported prevalence rates in the Asia Pacific region have been variable and ranged from 5% to 40% [14–17]. Clinical characteristics of our cohort were also consistent with current literature. There was a preponderance of NAFLD in males, echoing the findings from several population-based epidemiological studies [18–20]. Similarly, subjects with NAFLD were older and had higher BMIs, larger waist circumferences and greater frequency of observed metabolic risk factors [5,14,21]. Interestingly, while there was a significant difference in mean BMI between subjects with and without NAFLD, the BMI in both groups were below 25 kg/m^2^. Established data demonstrate that Asian populations have greater metabolic morbidity and mortality compared with Western populations at any given BMI level [22,23]. Hence, the consensus for lowering BMI cut-off points in Asian subjects, with a BMI more than 25 kg/m^2^ defining obesity in adult Asians [24]. Having said that, as with our study, NAFLD can occur in non-obese subjects even with the lower Asian BMI cut-offs. This phenomenon, termed ‘lean metabolic obesity’, carries increased risk of insulin resistance, metabolic syndrome and NAFLD despite normal BMI and has been more evidently demonstrated in Asian series [25–28]. As such, health care providers and patients alike need to be cognisant of the potential development of NAFLD in subjects with relatively low BMI if other associated risk factors are present and manage these patients appropriately.

With regard to attitudes towards NAFLD, the majority of our subjects had heard about NAFLD as a disease entity and knew that it was a potentially dangerous condition. However, the majority of subjects did not feel that they were at risk of having NAFLD. This held true even for subjects with one or more metabolic risk factors and also for those who satisfied criteria for metabolic syndrome (central obesity plus any two other metabolic risk factors). This suggests superficial awareness about NAFLD and lack of appreciation of the potential risks in vulnerable subjects. Indeed, other community-based surveys of attitudes and knowledge of NAFLD have shown relatively poor awareness about the disease [29,30]. Eighty-one percent of responders to a Hong Kong telephone survey perceived their knowledge of NAFLD to be inadequate, while 18% of responders to an American survey reported low awareness of NAFLD even in at-risk subjects [29,31]. The underestimation of risk may result in affected individuals remaining undiagnosed and presenting only in the later stages of symptomatic advanced disease. Admittedly, more subjects with metabolic syndrome felt that they were at risk of NAFLD compared with those without metabolic syndrome. However, it is difficult to interpret and draw firm conclusions as the number of subjects with metabolic syndrome was considerably low.

All things considered, our study findings advocate for better awareness of NAFLD through promotion and education of risk recognition in select populations. Interestingly, the majority of subjects were receptive to NAFLD screening. However, en masse screening for NAFLD should not be advocated; instead, screening should be promoted for at-risk individuals.

The potential limitations of our study include the cross-sectional design which allows evaluations of associations only. There could also be a possible selection bias as subjects were recruited from a public gastroenterology forum and could possibly be more knowledgeable about gastroenterological conditions or may have pre-existing medical conditions that prompted them to attend such a forum. In addition, subjects at these forums may be more pro-active in health-care seeking/screening behaviour. This possible selection bias may potentially overestimate the NAFLD impact compared with studying an unselected population. Nevertheless, this selection bias may be limited by initial screening to exclude subjects with pre-existing liver conditions. Furthermore, despite the limitations of our study, to our knowledge this study provides the first important epidemiological community data of NAFLD in Singapore and demonstrates a significant presence of NAFLD among the community. Singapore, as with the rest of Asia, has undergone rapid modernization with increasing affluence and a shift toward a more sedentary lifestyle and obesogenic dietary patterns. As such, metabolic diseases such as NAFLD can only continue to pose a significant public health and clinical burden. Our data provide a glimpse of the enormity of the problem.

Our study suggests a significant risk of NAFLD in the community, even among non-obese individuals. While the majority of subjects had heard about NAFLD, there was relative underestimation of perceived risk. Enhanced public education is warranted to improve understanding, and further exploration into the awareness and attitudes of NAFLD is needed to develop strategies for combatting this disease.
